# Associations between health-related social needs and colorectal cancer screening among United States Hispanic/Latino and non-Hispanic white adults

**DOI:** 10.1016/j.pmedr.2026.103670

**Published:** 2026-10-16

**Authors:** Isaiah Casarez, Humberto Parada, Matthew P Banegas, Benjamín Aceves

**Affiliations:** a San Diego State University Research Foundation, San Diego State University, San Diego, California, United States; b Division of Epidemiology and Biostatistics, School of Public Health, San Diego State University, San Diego, United States; c UC San Diego Health Moores Cancer Center, La Jolla, California, United States; d Division of Health Promotion and Behavioral Science, School of Public Health, San Diego State University, San Diego, United States; e Department of Radiation Medicine and Applied Sciences, School of Medicine, University of California, San Diego, United States

**Keywords:** Colorectal cancer screening, Health-related social needs, Clinician recommendation, Health disparities, National health interview survey

## Abstract

**Objective:**

To examine associations between five health-related social need (HRSN) domains and receipt of a clinician recommendation for colorectal cancer (CRC) screening among Hispanic/Latino and non-Hispanic White adults, and whether associations differed by race and ethnicity.

**Methods:**

We pooled 2021 and 2023 National Health Interview Survey data (United States) for 1477 Hispanic/Latino and 5413 non-Hispanic White adults aged 45–75 not up to date on screening. The outcome was a clinician screening recommendation in the past 12 months. Five HRSNs were examined: educational need, food insecurity, housing instability, medical financial hardship, and public cash assistance. Survey-weighted logistic regression estimated adjusted odds ratios (aOR) and 95% confidence intervals (CI), overall and by race and ethnicity.

**Results:**

Very low food security (aOR 1.62; 95% CI 1.09, 2.40) and medical financial hardship (aOR 1.28; 95% CI 1.09, 1.49) were associated with higher odds of recommendation. Among Hispanic/Latino adults, low (aOR 2.12; 95% CI 1.04, 4.32) and very low food security (aOR 2.86; 95% CI 1.31, 6.23) were associated with recommendation; the food-by-race/ethnicity interaction was not significant (*p* = 0.09).

**Conclusions:**

Health-related social needs were not uniformly associated with reduced recommendation; food insecurity predicted higher recommendation among Hispanic/Latino adults.

## Highlights

• Food insecurity was linked to more screening recommendation in Hispanic adults
• Health-related social needs did not uniformly lower screening recommendation
• Educational need was linked to lower odds of a screening recommendation
• The need-recommendation link was concentrated in Hispanic adults

## 1 Introduction

Colorectal cancer (CRC) remains a leading cause of cancer-related morbidity and mortality in the United States, with more than 150,000 new cases and over 50,000 deaths annually (American Cancer Society, 2026; Siegel et al., 2023). Among Hispanic/Latino adults, CRC is the second most frequently diagnosed cancer among men and the third most frequently diagnosed cancer among women (American Cancer Society, 2024). Colonoscopy and stool-based tests are effective screening tools for CRC prevention and early detection, and although screening uptake among United States adults aged 50–75 years has increased, substantial disparities persist among those under 50 years and among certain Hispanic/Latino populations, particularly individuals of Mexican origin (Sabatino et al., 2025; Boersma et al., 2024). We distinguish here between disparities in colorectal cancer incidence and mortality and disparities in the screening pathway itself; the present study concerns the screening-recommendation step rather than screening uptake or cancer outcomes.

Health-related social needs (HRSNs) including food insecurity, housing instability, and medical financial hardship are increasingly recognized as individual-level social determinants of health that constrain access to preventive care (Livingston et al., 2025). Hispanic/Latino adults experience a disproportionate burden of unmet HRSNs relative to non-Hispanic White adults, including higher prevalence of food insecurity, housing instability, and medical financial strain, which may limit both patient healthcare engagement and clinician decision-making around CRC screening (Livingston et al., 2025). Prior research has linked unmet HRSNs to reduced healthcare utilization among Hispanic/Latino adults ([Anonymous], 2023). However, few studies have examined HRSNs and CRC screening using comprehensive, multi-domain measures (Ebner et al., 2024; Wang, 2025), and fewer still have assessed whether these associations differ by race and ethnicity. This is important given evidence that Hispanic/Latino adults are significantly less likely than non-Hispanic White adults to receive clinician recommendations for CRC screening, a recognized driver of screening uptake (Sabatino et al., 2025; Wang, 2025). Additionally, cardiometabolic comorbidity burden, disproportionately elevated among Hispanic/Latino adults and associated with increased clinical contact frequency, may confound the association between HRSN and screening recommendation receipt. Because HRSNs are themselves upstream determinants of chronic disease, failing to account for comorbidities may conflate disease-driven care-seeking behavior with the independent effects of social adversity on clinician recommendation behavior; yet comorbidities have rarely been considered in this context ([Anonymous], 2023; Daviglus et al., 2012).

To address these research gaps, we used data from National Health Interview Survey (NHIS) to examine the associations between five HRSN domains including food insecurity, educational need, housing instability, medical financial hardship, and public assistance receipt and receipt of a clinician recommendation for colorectal cancer screening in the past year among United States adults aged 45–75 years without a history of cancer, adjusting for cardiometabolic comorbidity. We also examined whether these associations differed between Hispanic/Latino and non-Hispanic White adults. We hypothesized that greater unmet social needs would be associated with lower odds of recommendation receipt, and that associations would differ in magnitude and direction by racial/ethnic group reflecting differences in care setting, structural barriers, and social need profiles. The hypothesized statistical modification by race/ethnicity was not supported; the association was directionally concentrated among Hispanic/Latino adults but did not reach significance on formal testing.

## 2 Methods

### 2.1 Study design and population

This cross-sectional study used pooled data from the 2021 and 2023 NHIS, a multistage, stratified, clustered probability sample of the non-institutionalized United States civilian population. The analytic cohort included adults aged 45–75 years who self-identified as Hispanic/Latino or non-Hispanic White, reported no prior history of cancer, and responded to the CRC screening recommendation item (unweighted *n* = 6890; weighted *N* = 29.4 million), including 5413 non-Hispanic White adults (weighted *N* = 21.7 million) and 1477 Hispanic/Latino adults (weighted *N* = 7.7 million). By NHIS design, the recommendation item was administered only to adults not currently up to date with colorectal cancer screening on any modality, that is, those who had never had a colonoscopy or had one more than 10 years previously, never had a sigmoidoscopy or computed tomographic colonography or had one more than 5 years before, and never had a stool-based test within its recommended interval. Adults who were up to date under modality specific timelines were skipped and are therefore not a part of the study population. Because these individuals are excluded by design, prior screening modality cannot bias recommendation frequency, and the analytic population is best understood as adults who are due or overdue for screening. Of the 59,004 sample adults in the pooled 2021 and 2023 National Health Interview Survey, we excluded 28,943 outside the 45 to 75 year age range, 4554 with a history of cancer, and 5033 not identifying as Hispanic/Latino or non-Hispanic White, and 13,584 who were not administered or did not respond to the recommendation item, yielding 6890 analytic respondents; adjusted models used the 6507 respondents with complete covariate data.

### 2.2 Measures

The primary outcome was receipt of a clinician recommendation for CRC screening in the past 12 months. Participants were asked, “In the past 12 MONTHS, did a doctor or other health professional RECOMMEND that you be tested to look for problems in your colon or rectum?” Response options included “yes,” “no,” and “don't know or refused.” Among age-eligible, cancer-free adults, item nonresponse to the recommendation question was low (1.3%). The large proportion of individuals that were not asked the question reflects the skip logic described above, not missing data.

Five Health-related social needs (HRSNs) were examined, including food insecurity, educational need, housing instability, medical financial hardship, and public assistance receipt. Educational need, housing instability, medical financial hardship, and public cash assistance were each operationalized as binary indicators. Food insecurity was assessed using the NHIS 30-day food security screener under both four-category (high, marginal, low, very low) and three-category (high/marginal, low, very low) classifications, with the latter serving as a sensitivity analysis on the placement of marginal food security, which the United States Department of Agriculture (USDA) classifies as food secure but may reflect meaningful unmet need. Educational need was defined as less than a high school diploma. In the initial analysis this domain also incorporated employment status; however, examination of reasons for not working showed that the majority of adults classified as not working were retired or reported a disability rather than experiencing unmet employment need, so the domain was redefined as educational need alone, consistent with its use as a social risk indicator in prior work ([Anonymous], 2025). Housing instability was defined using a single measure available in both survey years, receipt of government housing assistance among renters, with homeowners classified as housing stable. A housing-cost burden item was fielded in 2023 but not in 2021; to preserve a consistent definition across years, we did not incorporate cost burden. This harmonized measure is therefore a narrow indicator of housing need. Medical financial hardship was defined as problems paying medical bills in the past 12 months or worry about doing so. Public assistance receipt was based on public cash assistance income in the past 12 months.

Sociodemographic covariates included age (continuous), sex (male or female), nativity (born in the United States or outside the United States), income-to-poverty ratio (<100%, 100–199%, 200–399%, ≥400%), insurance status (no or yes), census region (Northeast, Midwest, South, West), urbanicity (large metro, medium/small metro, non-metro regions), and survey year (2021 or 2023). Cardiometabolic burden was operationalized as a count of seven conditions (hypertension, high cholesterol, diabetes, myocardial infarction, stroke, chronic obstructive pulmonary disease, and body mass index), categorized as 0, 1–2, or 3 and greater.

### 2.3 Statistical analysis

We fit survey-weighted logistic regression models with quasibinomial variance estimation, including all HRSN domains and adjusting for all sociodemographic covariates and cardiometabolic burden. Multicollinearity among HRSN predictors was assessed via pairwise phi coefficients and variance inflation factors (VIFs). All phi coefficients were below 0.30 and VIFs ranged from 1.04 to 1.21, indicating no meaningful collinearity. Models were fit in the overall analytic sample and separately by race/ethnicity. Adjusted odds ratios (aOR) and 95% confidence intervals (CI) were estimated. Adjusted models were fit using complete cases; 383 participants (5.5%) with missing data on one or more covariates were excluded, leaving 6507 in the adjusted analysis. We tested food insecurity-by-race/ethnicity interaction terms. All analyses incorporated complex NHIS survey design features with modified pooled two-year weights. In sensitivity analysis, we re-specified food security as a binary indicator (food insecure = low or very low; food secure = high or marginal, per the USDA cut point) to assess robustness to the ordinal specification. Additionally, we adjusted the primary model for recent preventive contact, defined as a wellness or general check-up visit within the past 12 months (constructed from the survey visit-type and visit-recency items), to assess whether associations reflected differential access to preventive encounters rather than social need. All analyses were conducted in R (version 4.5.2) using the survey package.

## 3 Results

Survey-weighted descriptive characteristics are presented in Table 1. The weighted mean age was 54.7 years (SE = 8.1) and approximately 47.5% were female. Most adults resided in large metropolitan areas (50.8%). Hispanic/Latino adults had substantially lower household incomes than non-Hispanic White adults (26% vs. 11.3% below 100% of the federal poverty level (FPL); 20.5% vs. 48.8% at or above 400% FPL) and were more likely to live in large metropolitan areas (64.0%) and in the South (42.2%) or West (40.1%) Census regions.

**Table 1:** Weighted demographic characteristics and health-related social needs by race and ethnicity among United States Hispanic/Latino and non-Hispanic White adults aged 45–75 not up to date on colorectal cancer screening, National Health Interview Survey, 2021 and 2023.

Characteristic	Non-Hispanic White	Hispanic	Overall
	*N* = 21,659,897^1^	*N* = 7,708,045^1^	*N* = 29,367,943^1^
	%	%	%
Age, mean (SE), y	55.2 (8.2)	53.4 (7.5)	54.7 (8.1)
Sex			
Male	52.6	52.2	52.5
Female	47.4	47.8	47.5
Nativity			
U.S Born	93.7	26.8	76.3
Non-US Born	6.3	73.2	23.7
Income-to-Poverty Ratio Quartile			
<100% FPL	11.3	26.0	15.1
100–199%	18.7	33.1	22.5
200–399%	21.2	20.4	21.0
≥400%	48.8	20.5	41.4
Insured			
No	8.7	27.2	13.5
Yes	91.3	72.8	86.5
Region			
Northeast	18.4	10.3	16.2
Midwest	26.7	7.5	21.7
South	36.3	42.2	37.8
West	18.6	40.1	24.3
Population Density			
Large Metro	46.2	64.0	50.8
Medium/Small Metro	33.2	29.9	32.3
Nonmetro	20.6	6.2	16.8
Cardiometabolic Burden			
0 Conditions	35.6	35.4	35.5
1–2 Conditions	48.9	50.6	49.3
3 or greater Conditions	15.7	14.0	15.2
Individual health-related social needs^a^			
Education Need	6.9	41.2	15.8
Food Insecurity	6.5	12.3	8.0
Housing Instability	4.8	8.2	5.7
Medical Financial Strain	49.8	67.9	54.6
Public Assistance	3.2	4.4	3.5

1 Mean (SD); n (%)

a Among respondents to each HRSN

### 3.1 Associations between health-related social needs and colorectal cancer screening recommendation

Adjusted logistic regression model results are presented in Table 2. Among the five HRSN domains examined, very low food security and medical financial hardship were independently associated with higher odds of receiving a CRC screening recommendation, while educational need, housing instability, and public assistance receipt were not significantly associated with recommendation receipt. Very low food security was statistically significant in both the four-category (aOR 1.62; 95% CI 1.09, 2.40) and three-category (aOR 1.68; 95% CI 1.14, 2.48) models. Low and marginal food security was not statistically significant in either model. Medical financial hardship was independently associated with higher odds of recommendation receipt (aOR 1.28; 95% CI 1.09, 1.49), consistent across both food security model versions. Among sociodemographic predictors, foreign-born status was associated with lower odds of recommendation receipt (aOR 0.70; 95% CI 0.55, 0.90), as was residence in the South relative to the Northeast (aOR 0.67; 95% CI 0.52, 0.85). Higher income (≥400%FPL) was associated with greater odds of recommendation receipt (aOR = 1.52; 95%CI:1.14–2.03). Cardiometabolic burden showed a strong dose-dependent association with recommendation receipt, with adults with 1–2 conditions having 59% higher odds of recommendation receipt relative to those with no conditions (aOR 1.59; 95% CI 1.31, 1.92), and those with three or more conditions nearly 2.7 times the odds (aOR 2.74; 95% CI 2.17, 3.45). Health insurance coverage was the strongest individual predictor across all models (aOR 3.18; 95% CI 2.21, 4.58). Recent preventive contact was strongly associated with recommendation receipt (aOR 2.36; 95% CI 1.95, 2.86). Adjusting for recent preventive contact did not lessen the food security association;, the very low food security estimate was 1.58 (95% CI 1.07, 2.35) without and 1.70 (95% CI 1.14, 2.54) with the preventive-visit term, indicating that differential access to preventive encounters does not account for the association. Findings were likewise unchanged after adjusting for any prior colorectal test (ever vs never) in this not-up-to-date population (very low food security aOR 1.62; 95% CI 1.09, 2.40), with prior testing itself associated with lower odds of a new recommendation (aOR 0.77; 95% CI 0.63, 0.95). In a sensitivity analysis using a binary food security indicator (insecure vs secure), the association was consistent in direction and significance with the primary ordinal model (pooled aOR 1.55; 95% CI 1.14, 2.11, and aOR 1.47; 95% CI 1.08, 1.99 with additional adjustment for cardiometabolic burden), with a smaller point estimate as expected when the low and very low categories are combined.

**Table 2:** Adjusted associations between health-related social needs and receipt of a clinician recommendation for colorectal cancer screening among United States Hispanic/Latino and non-Hispanic White adults aged 45–75 not up to date on screening, National Health Interview Survey, 2021 and 2023.

	Pooled	Non-Hispanic White	Hispanic/Latino
	aOR (95% CI)	aOR (95% CI)	aOR (95% CI)
Health-Related Social Needs			
Food Insecurity (Ref: High)			
High	Ref (American Cancer Society, 2026).00)	Ref (American Cancer Society, 2026).00)	Ref (American Cancer Society, 2026).00)
Marginal	0.74 (0.51, 1.09)	0.85 (0.56, 1.29)	0.45 (0.18, 1.16)
Low	1.29 (0.85, 1.94)	0.97 (0.56, 1.29)	2.12 (1.04, 4.32)
Very low	1.62 (1.09, 2.40)	1.31 (0.84, 2.03)	2.86 (1.31, 6.23)
Educational Need (Ref: No Need)	0.58 (0.43, 0.77)	0.61 (0.41, 0.90)	0.62 (0.38, 0.99)
Housing Instability (Ref: Stable)	1.04 (0.68, 1.57)	1.16 (0.70, 1.90)	0.75 (0.32, 1.75)
Public Assistance (Ref: None)	0.74 (0.47, 1.17)	0.85 (0.51, 1.43)	0.37 (0.12, 1.11)
Selected Covariates			
Insured (Ref: Uninsured)	3.18 (2.21, 4.58)	3.20 (2.09, 4.91)	2.86 (1.44, 5.68)
Cardiometabolic Burden: 1–2 conditions	1.59 (1.31, 1.92)	1.66 (1.34, 2.04)	1.27 (0.81, 1.98)
Cardiometabolic Burden: 3 conditions or greater	2.74 (2.17, 3.45)	2.71 (2.10, 3.51)	2.87 (1.68, 4.88)

aOR, adjusted odds ratio; CI, confidence interval; HRSN, health-related social need; Ref, reference category. Estimates are from a survey-weighted logistic regression model adjusting for age, sex, nativity, income-to-poverty ratio, insurance, census region, urbanicity, survey year, educational need, housing instability, medical financial hardship, public cash assistance, and cardiometabolic burden. The reference category for each variable is shown as 1.00. The food insecurity-by-race and ethnicity interaction was assessed with a joint Wald test (F = 2.14; degrees of freedom = 3 and 1135; *p* = 0.09).

### 3.2 Race and ethnicity-stratified analyses

In race-stratified models, food security associations were pronounced among Hispanic adults, both low (aOR 2.12; 95% CI 1.04, 4.32) and very low food security (aOR 2.86; 95% CI 1.31, 6.23), and associated with higher odds of recommendation receipt, consistent across models. No significant food security associations were observed among non-Hispanic White adults. Medical financial hardship was statistically significant among non-Hispanic White adults (aOR 1.26; 95% CI 1.06, 1.49) but was not statistically significant among Hispanic adults (aOR 1.56; 95% CI 0.98, 2.48), although the estimate was in the same direction. Foreign-born status was associated with lower odds of recommendation receipt among Hispanic adults (aOR 0.71; 95% CI 0.47, 1.07) but not among non-Hispanic White adults. Housing instability was inversely associated with recommendation receipt among Hispanic adults (aOR 0.75; 95% CI 0.32, 1.75), but not among non-Hispanic white adults. The food-by-race/ethnicity interaction was not statistically significant (joint Wald test F = 2.14; df = 3, 1135; *p* = 0.09); accordingly, the stratified estimates below describe where the association was concentrated and should not be interpreted as evidence of statistical effect modification. Notably, nonmetro residence was inversely associated with recommendation receipt among Hispanic adults (aOR 0.23; 95% CI 0.06, 0.82) but not among non-Hispanic White adults. Cardiometabolic burden was a strong predictor of recommendation receipt in both groups.

## 4 Discussion

Among the five HRSN domains examined, very low food security and medical financial hardship were independently associated with receipt of CRC screening recommendation, while educational need, housing instability, and public assistance receipt were not. Rather than reflecting direct pathways from social need to preventative care, these associations may reflect differential healthcare contact through safety-net settings, federally qualified health centers, and community health programs, where HRSN screening is more routinely integrated into care delivery and clinicians may be more attuned to preventative care priorities (Fraze et al., 2019). This pattern raises whether higher-need adults are less likely to be offered screening or less likely to obtain it. Among this overdue population, greater need was associated with more, not less, recommendation, pointing away from a failure to offer and toward downstream barriers to completing screening.

Race-stratified analyses indicated that among Hispanic/Latino adults, food insecurity was associated with recommendation receipt in a dose-dependent manner, consistent with evidence that safety-net utilization among food insecure populations is disproportionately higher in this group (Shin et al., 2015). In the Hispanic/Latino stratum, housing instability was not significantly associated with recommendation (aOR 0.75; 95% CI 0.32, 1.75). Nonmetropolitan residence showed lower odds of recommendation (aOR 0.23; 95% CI 0.06, 0.82), though this estimate derives from a small number of respondents (Kushel et al., 2006). The nonmetropolitan finding is particularly notable given sparse safety-net infrastructure in rural areas and documented primary care shortages serving Hispanic/Latino communities (Kao et al., 2025; Probst et al., 2004). In the overall sample, foreign-born nativity was independently associated with lower odds of recommendation receipt (aOR 0.70; 95% CI 0.55, 0.90); in the Hispanic/Latino stratum the estimate was directionally similar but did not reach significance (aOR 0.71; 95% CI 0.47, 1.07). This pattern is consistent with prior evidence of structural barriers among immigrant populations including language, documentation status, and insurance eligibility constraints (Vargas Bustamante et al., 2012; Derose et al., 2009). The subgroup difference should therefore be interpreted as hypothesis-generating and may reflect limited power in the smaller Hispanic/Latino stratum. Contact with institutions may carry immigration-related consequences, a climate associated with reduced use of health and social services, including preventive care [REF]. Although our cross-sectional data cannot identify specific mechanisms, these findings are consistent with the sociopolitical environment surrounding immigration acting as an upstream determinant of preventive-care engagement.

Cardiometabolic burden had a strong dose-dependent association with recommendation receipt in both groups, consistent with the well-documented relationship between comorbidity and increased clinical encounter frequency (Barnett et al., 2012). Because HRSNs are upstream determinants of chronic disease, failure to adjust for comorbidity risks conflating social adversity with disease-driven care-seeking, a primary concern among Hispanic/Latino adults, who carry disproportionately high rates of diabetes, hypertension, obesity, and whose comorbidity-driven encounters may serve as touchpoints for screening recommendation delivery (Daviglus et al., 2012).

Several limitations should be noted. The cross-sectional design precludes causal inference, and the direction of the food insecurity-recommendation association cannot be interpreted as a direct causal pathway without longitudinal data. The outcome captures clinician recommendation, not screening completion, limiting conclusions about downstream behavior. The Hispanic/Latino stratum was relatively small, resulting in imprecise estimates for less prevalent HRSNs and interaction tests that were underpowered. Housing instability measurement differed across survey years due to item availability, which may introduce misclassification. Because every analyzed adult was not up to date on screening, a positive association between social need and recommendation is most consistent with a recommendation-to-uptake gap, need-associated adults being told to screen but not yet completing it, rather than a failure to be offered screening. Within the Hispanic/Latino sample, only 30 adults reported Black race, too few to support stable subgroup estimation, and a large number reported “other or multiple” race, reflecting the known difficulty of identifying Afro-Latino adults from standard race and ethnicity items in the NHIS. We were therefore unable to examine associations specific to Afro-Latino adults, an important direction for future work using data designed to capture these distinctions. The survey ascertains binary sex rather than gender identity, so we could not examine gender-related differences. The data contain no information on clinician characteristics, preventing examination of patient-clinician racial or ethnic concordance, which prior work links to greater use of preventive care (Meyers et al., 2025). Access to specialty and gastroenterology care, particularly in low-resource settings, was also unmeasured and may shape whether a recommendation is completed. Last, residual confounding by healthcare setting type, language of care delivery, and acculturation may be possible.

## 5 Conclusions

These findings indicate that HRSNs are not uniformly associated with reduced preventive care receipt. Rather, the direction and magnitude depend on the specific need domain, population subgroup, and likely the care setting in which the patients are engaged. Efforts to improve CRC screening equity among Hispanic/Latino adults should address not only food insecurity but also structural barriers associated with housing instability, rural residence, and immigrant status, which were associated with lower odds of recommendation receipt in this group. These results suggest that safety-net settings serving food-insecure Hispanic/Latino patients may represent a strategic leverage point for integrating CRC screening promotion.

## Declaration of generative AI and AI-assisted technologies in the manuscript preparation process

During the preparation of this work, the author(s) used Claude (Anthropic) to improve the grammar and sentence structure of the manuscript. The author(s) reviewed and edited the content as needed and take full responsibility for the content of the published article.

## CRediT authorship contribution statement

**Isaiah Casarez:** Writing – review & editing, Writing – original draft, Software, Formal analysis, Data curation. **Humberto Parada:** Writing – review & editing, Writing – original draft, Supervision, Methodology, Conceptualization. **Matthew P Banegas:** Writing – review & editing, Writing – original draft, Supervision, Methodology, Conceptualization. **Benjamín Aceves:** Writing – review & editing, Writing – original draft, Supervision, Methodology, Conceptualization.

## Ethics approval

This study used publicly available, de-identified data from the National Health Interview Survey. No institutional review board approval was required.

## Ethics declaration

The authors have NOT obtained informed consent from participants or their legal representatives. This study was a secondary analysis of fully de-identified, publicly available data from the National Health Interview Survey, conducted by the National Center for Health Statistics (NCHS). The authors had no contact with participants and no access to identifiable information, and individuals cannot be identified from the public-use data files. Under the United States Common Rule (45 CFR 46), secondary research using de-identified data of this kind does not constitute human subjects research; therefore, individual informed consent for this analysis was not required, and institutional review board approval was not sought. Informed consent for the original data collection was obtained by NCHS at the time of the survey.

This study was performed in compliance with relevant laws, regulatory frameworks and guidelines where the research took place. This study was conducted in accordance with The analysis used public-use NHIS data in accordance with the NCHS data use terms and applicable US regulations. Ethics committee approval was not required under relevant laws and institutional guidelines. Secondary analysis of de-identified, publicly available data is not human subjects research under the US Common Rule (45 CFR 46), so IRB review was not required.

## Funding

This research received no specific grant from any funding agency in the public, commercial, or not-for-profit sectors.

## Declaration of competing interest

The authors declare that they have no known competing financial interests or personal relationships that could have appeared to influence the work reported in this paper.

## Data Availability

The data used in this study are publicly available from the National Center for Health Statistics. The National Health Interview Survey public-use data files and documentation can be accessed at https://www.cdc.gov/nchs/nhis/. The authors did not generate any new data.
